# Low Dietary Intakes of Essential Nutrients during Pregnancy in Vietnam

**DOI:** 10.3390/nu10081025

**Published:** 2018-08-06

**Authors:** Cong Luat Nguyen, Dong Van Hoang, Phung Thi Hoang Nguyen, Anh Vo Van Ha, Tan Khac Chu, Ngoc Minh Pham, Andy H Lee, Dat Van Duong, Colin W Binns

**Affiliations:** 1 National Institute of Hygiene and Epidemiology, Hanoi 100000, Vietnam; luatcong.nguyen@postgrad.curtin.edu.au (C.L.N.); hvd@nihe.org.vn (D.V.H.); 2 School of Public Health, Faculty of Health Sciences, Curtin University, Perth, WA 6102, Australia; nthphungytcc@ump.edu.au (P.T.H.N); anhhvv@pnt.edu.vn (A.V.V.H); cktan@hpmu.edu.vn (T.K.C.); minh.pn@tnu.edu.vn (N.M.P); Andy.Lee@curtin.edu.au (A.H.L.); 3 Department of Nutrition and Food, Faculty of Public Health, University of Medicine and Pharmacy, Ho Chi Minh City 700000, Vietnam; 4 Department of Environmental and Occupational Health, Pham Ngoc Thach University of Medicine, Ho Chi Minh City 700000, Vietnam; 5 Department of Epidemiology, Faculty of Public Health, Hai Phong University of Medicine and Pharmacy, Hai Phong 180000, Vietnam; 6 Department of Epidemiology, Faculty of Public Health, Thai Nguyen University of Medicine and Pharmacy, Thai Nguyen 250000, Vietnam; 7 Department of Sexual & Reproductive Health, United Nations Population Fund, Hanoi 100000, Vietnam; dat@unfpa.org

**Keywords:** dietary intake, food intake, nutrients, macronutrients, micronutrients, pregnancy, Vietnam

## Abstract

Inadequate intake of nutrients during pregnancy has been associated with poor pregnancy and infant outcomes; however, evidence remains limited in low-resource settings in Asia. This paper assessed food, macronutrient, and micronutrient intakes among 1944 Vietnamese pregnant women. Dietary information was collected via an interviewer-administered food frequency questionnaire, and nutrient intakes were estimated using the Vietnamese food composition tables. The levels of nutrient intakes were evaluated against the Vietnamese recommended nutrient intakes (RNI) for pregnancy. The diet profiles were reported as means and percentages. The average daily food intakes across socio-demographic factors were compared using ANOVA, with adjustment for multiple comparisons by the Tukey–Kramer test. Rice, fruits, and vegetables were the main food sources consumed. The mean energy intake was 2004 kcal/day with 15.9%, 31.8%, and 52.2% of energy deriving from proteins, fats, and carbohydrates, respectively. Just over half of the women did not meet the RNI for total energy intake. The intakes of essential micronutrients including folate, calcium, iron, and zinc were below the RNI, and almost all pregnant women failed to meet the recommendations for these micronutrients. The associations of maternal age, education, and pre-pregnancy body mass index with nutrient intakes varied across the nutrient subgroups. Targeted programs are needed to improve nutrient intakes in Vietnamese pregnant women.

## 1. Introduction

Maternal diet during pregnancy plays a vital role in maternal and child health. Both undernutrition and overnutrition during pregnancy are associated with an increased risk of adverse pregnancy outcomes [1,2], obesity, and chronic disease in adult life [3,4]. While overnutrition and obesity during pregnancy are common in developed countries [5,6], undernutrition among pregnant women including low intakes of macro- and micronutrients remains a challenge in developing nations [7,8]. A recent review indicated that the mean intakes of macronutrients and of the most essential micronutrients during pregnancy in low- and middle-income countries, including folate, iron, calcium, and zinc, were below the recommendations of the Food and Agriculture Organization of the United States and World Health Organization (WHO) [7]. Poor maternal nutrition has been documented in some Asian countries including China, India, Bangladesh, and Thailand [9,10,11,12,13,14,15].

Evidence suggests that inadequate intakes of macro- and micronutrients during pregnancy may cause adverse health outcomes in both mothers and their infants. Energy deficiencies and protein restriction are linked to low birth weight [16], while a high dietary glycemic load is associated with an increased risk of gestational diabetes mellitus [17]. A low vitamin D status may lead to low birth weight, increased childhood adiposity, or poor foetal skeletal development [18,19,20]. Folate deficiency during the periconceptional period is associated with an increased risk of neural tube defect [21] or congenital heart defects [22], and an inadequate calcium intake is known to elevate the risk of pre-eclampsia and maternal deaths [23]. Low intakes of important minerals such as iron and zinc have been associated with anaemia, low birth weight, preterm delivery [24], congenital anomalies, and fetal growth retardation [25]. As such, information about maternal diet during pregnancy would be useful for developing appropriate interventions to improve the health and well-being of mothers and children.

Although Vietnam has made significant progress in maternal nutrition, deficiencies of macro- and micronutrients in adults and women of reproductive age (WRA) are still a public health issue [26,27,28,29,30]. According to the most recent national nutrition survey in 2009, 20% and 70% of the Vietnamese adults did not meet the recommendations for energy and most micronutrients intakes, respectively [26]. The prevalence of inadequate intakes of most micronutrients among WRA was high, as shown for zinc (67%), vitamin B12 (63.8%), folate (54.3%), vitamin B2 (40.4%), vitamin A (27.1%), and iron (24.8%) [28,29]. Given that approximately 1.5 million babies are born every year in Vietnam [31], with nearly 12% of preterm births [32] and nearly 25% of children under five years of age being stunted [33], investigating the nutrition profiles of women during pregnancy is envisaged to uncover elements for future research and practice. However, to the best of our knowledge, there has been no study on maternal dietary intakes during pregnancy in Vietnam. The objective of this study was to assess the food, macronutrient, and micronutrient intakes of Vietnamese pregnant women.

## 2. Materials and Methods

### 2.1. Study Design and Population

This study used baseline data of a large prospective cohort study in Vietnam. The details of the study protocol have been published elsewhere [34]. Briefly, eligible pregnant women were recruited from six hospitals at prenatal care visits during early pregnancy in three metropolitan cities of Vietnam, namely, Ha Noi, Hai Phong, and Ho Chi Minh City. The participants were informed of the study purpose and procedure before they were asked for informed consent. During August 2015 and July 2016, the eligible participants were invited to attend a face-to-face interview at 24–28 weeks of gestation to provide information on their lifestyle, including dietary intake, physical activity, smoking, socio-demographics, and medical history.

### 2.2. Dietary Assessment

Information on dietary intakes was collected by trained interviewers using a modified version of the Food Frequency Questionnaire (FFQ) that had been validated for use in Vietnamese adults [35]. The FFQ includes a list of 119 common food and beverage items which are organized into 18 groups: (1) alcohol; (2) coffee; (3) tea; (4) fruit juices and soft drinks; (5) soybean products; (6) vegetables; (7) fruits; (8) sweet desserts; (9) cereals; (10) red meat; (11) poultry; (12) offal; (13) fish & seafood; (14) eggs; (15) preserved food; (16) dairy products; (17) seasoning; (18) supplement. For each food item, the participants were asked to report the frequency (times per day, week, or month) and the quantity (number of standardized servings each time) since they became pregnant. Pictures (full size) of commonly used tableware were compiled and used during the interview to determine the average portion sizes and average number of servings per meal. Photographs of the types and amounts of food items, such as a set of spoons, cups, and bowls, were shown to the participants to aid portion size estimation.

The amounts of foods (e.g., meat, fruits, and vegetables) consumed were estimated using standardized portion sizes and converted into in grams per day. The daily intakes of macronutrients (carbohydrates, proteins, fats, and calories) and micronutrients (vitamins and minerals) were calculated using an ad-hoc computer algorithm, by referring to the Vietnamese food composition tables [36]. For calculating each nutrient intake for an individual, the consumption of a single food or food groups was multiplied by the corresponding average nutrient content; we then summed all the amounts from the previous calculations to achieve the total nutrient intake, as shown in the following equation: Intake (i) = c × cereal nutrient (i) + v × vegetable nutrient (i) + f × fruit nutrient (i) + s × soy nutrient (i) + m × meat nutrient (i) + sf × seafood nutrient (i) + e × egg nutrient + d × dairy nutrient + sw × sweet nutrient + b × beverage nutrient (‘i’ denotes each nutrient, and ‘c’, ‘f’, ‘s’, ‘m’, ‘sf’, ‘e’, ‘d’, ‘sw’, and ‘b’ stand for intakes of cereals, vegetable, fruit, soy foods, red meat and poultry, fish and seafood, egg, dairy, sweet desserts, and fruit juice or soft drinks, respectively). Subgroup classifications of fruits and vegetables were presented according to the part of plant consumed [37]. The recommended nutrient intakes (RNI) from the latest National Guidelines on Nutrition for Pregnant Women and Breastfeeding Mothers were used as reference values [38]. We adopted the national recommendation of total energy intake for women with light physical activity during pregnancy because approximately 71% of the participants engaged in light physical activity. The bioavailability levels of iron and zinc were assumed on the basis of the national guidelines on recommended nutrient intakes (RNI) for pregnant women used as a standard reference [38]. A high bioavailability of iron was defined as the intake of vitamin C >75 mg/day (183.4 mg/day in our study), while a medium bioavailability of zinc was defined as a moderate intake of proteins from animals or fish. Intakes below the RNI were considered inadequate. Since most vitamin D is synthesized endogenously, the data from the food sources is presented, but no comments on adequacy can be made. The sources of folate in the diet vary considerably in bioactivity, and, while data are presented for protection against neural tube defects, supplementation with folic acid is recommended for all pregnant women [39].

### 2.3. Assessment of Other Variables

Socio-demographic characteristics, including age, marital status, occupation, education, parity, and medical history were collected through a structured interview. Age was divided into four groups: <25, 25–29, 30–34, and ≥35 years old. Education was categorized into three categories based on the highest grade level completed: less than high school, high school, and further than high school. Pre-pregnancy body mass index (BMI) was calculated by dividing the weight obtained from medical records by the squared height measured at the baseline interview and expressed in kg/m^2^. Pre-pregnancy BMI was classified into three categories: underweight (<18.5 kg/m^2^), normal (18.5 ≤ BMI < 23.0 kg/m^2^), and overweight (≥23.0 kg/m^2^) according to the BMI cutoff for the Asia population [40]. Smoking status (active and passive smoking) was elicited using the WHO STEPS questions [41]. Passive smoking was defined as exposure to tobacco smoke at home or workplaces.

### 2.4. Statistical Analyses

Women who had an implausible total energy intake (<500 or >3500 kcal per day) were excluded from statistical analyses [42]. Descriptive analyses were used to report the socio-demographic characteristics of the study sample. The total daily intakes of food groups, macronutrients, and micronutrients are presented as mean ± standard deviation (SD). Differences in nutrient intakes across categories of age group, education level, and pre-pregnancy BMI were examined using ANOVA tests. For foods that showed a statistically significant test statistic (*F* statistic) for ANOVA (e.g., *p* < 0.05), we proceeded with the Tukey–Kramer test [43] to identify which specific groups had statistically significantly different means from one another. Energy and nutrient intakes were compared with the RNI for Vietnamese pregnant women to estimate the prevalence of adequate nutrient intakes [38]. The prevalence of adequacy for macronutrient intakes was determined as the proportion of participants with observed intakes that met, at a minimum, the following recommendations: 13–20% of energy from proteins, 20–30% of energy from total fat, and 55–65% of energy from carbohydrates. The corresponding data for micronutrients was defined as the prevalence of individuals with observed intakes that achieved the minimum or single values of the RNI [44]. In addition, energy-adjusted nutrient intakes were calculated using the density method (amount of nutrient intake per 1000 kcal of energy) [45]. All analyses were performed using the R Statistics software version 3.3.3 (R Foundation for Statistical Computing, Vienna, Austria) [46].

### 2.5. Ethics Approval

The study protocol was approved by the Human Research Ethics Committees of Curtin University in Australia (HR32/2015) and Hai Phong University of Medicine and Pharmacy in Vietnam (No. 05/HPUMPRB/2015). Written consent forms were obtained from all participants.

## 3. Results

### 3.1. Socio-Demographic Characteristics of the Study Participants

Of the 2248 eligible women approached, 2030 (90.3%) consented to participate in the study. Among them, 86 women were excluded from the study because of implausible total energy intakes, giving a final sample of 1944 women for analysis. The individual characteristics of these participants are presented in Table 1. The age ranged from 18 to 48 years, with a mean of 27.6 years of age (SD = 5.3). Nearly two-thirds of participants completed high school or higher education. The study sample had a mean pre-pregnancy BMI of 20.2 kg/m^2^ (SD = 2.5), and the prevalence of underweight and overweight were 25.4% and 12.9%, respectively. No women smoked during pregnancy, but over half of them were exposed to passive smoking. Approximately one in eight women drank alcohol during pregnancy.

### 3.2. Food Intake

Table 2 summarizes the mean daily food intakes (g) among pregnant women. Overall, cereals were the most common foods (682.8 g/day), followed by fruits (315.0 g/day) and vegetables (240.7 g/day). Rice was the main food source of cereals (85.9%), while banana was the most commonly consumed fruit (63.4 g/day). Leafy vegetables, fruit-vegetables, roots and tubers were the main types of vegetables consumed (89.0%). The daily intakes of poultry, eggs, fish and seafood, red meat, and soy products were low, ranging from 17.6 to 54.8 g/day. Very low intakes of alcohol, coffee, and tea during pregnancy were reported.

Table 3 shows the average daily intakes of foods and beverages together with multiple comparisons across age groups, educational levels, and pre-pregnancy BMI levels. In general, older women consumed more vegetables and fish and seafood than their younger counterparts, whilst young women consumed more cereals, fruits, red meat, poultry, eggs, sweet dessert, fruit juices and soft drinks than the older ones. There were statistically significant differences in the mean intakes of the aforementioned food items (except for fruits) between participants aged ≥35 years and those under 25 years of age (*p* < 0.05). The mean intakes of red meat, poultry, eggs, and fruit juices and soft drinks were significantly higher in more educated women, especially women with post-high school education. Overweight women ate soy products and fish and seafood more frequently but consumed less cereals, poultry, and eggs than those with a lower pre-pregnancy BMI. They had significantly lower mean intakes of cereals and poultry than pre-pregnancy underweight women. Meanwhile, their average intakes of soy products and fish and seafoods were significantly greater than those of underweight and normal-weight women before pregnancy, respectively.

### 3.3. Nutrient Intake

Table 4 presents the nutrient intakes among the participants, including energy, macronutrients, micronutrients, and comparisons with the RNI. The mean total energy intake was 2004 kcal/day (SD = 625), with 15.9%, 31.8%, and 52.2% of energy coming from proteins, fats, and carbohydrates, respectively. Overall, the energy intake met the national RNI, but only approximately half of the women achieved the recommendation. The mean intakes of proteins and fat were higher than the reference values, with the majority of women achieving the requirements.

The mean intakes of several micronutrients were above the RNI, whereas the intake levels of some important nutrients for optimal reproductive health, such as folate, calcium, iron, and zinc, were much lower than the RNI. Almost all participants did not meet the RNI for iron and calcium. Very few women had adequate intakes of folate (15.4%) and zinc (18.0%) before supplementation. The intake levels of different B vitamins not reaching the Vietnam RNI (not including folate) varied from 11.5% to 62.0%. The proportions with inadequate intakes of vitamin A and vitamin C were 41.3% and 27.7%, respectively. Most participants met the RNI for magnesium, selenium, phosphorus, and manganese.

Associations between nutrient intakes and selected socio-demographic characteristics including maternal age, educational level, and pre-pregnancy BMI are shown in Supplementary Table S1. In brief, older women consumed less energy, macronutrients, B group vitamins, iron, and zinc than younger women. In contrast, educated women consumed more proteins, B group vitamins, iron, and zinc than their counterparts with a lower education level. Overweight women consumed less energy and lower levels of almost all macro- and micronutrients than women with a lower pre-pregnancy BMI.

## 4. Discussion

This is the first large-scale study to report a comprehensive profile of the diet of pregnant women in Vietnam. It found that rice, fruits, and vegetables were the major food sources of energy, with approximately half of the women meeting the RNI for total energy intake. The data also showed that almost all pregnant women did not meet the RNI for some essential micronutrients, including folate, calcium, iron, and zinc.

Our finding of rice being the main food source is consistent with previous studies among pregnant women in China, India, and Thailand [10,13,47]. Despite the lack of dietary information during pregnancy in Vietnam for a direct comparison, rice was also the staple food in 1985 and 2010 in Vietnam according to nationally representative nutrition surveys [27]. However, the amount of rice intake in our study was much higher than in prior research [10,13,27,47]. Variations in the intake levels of fruits and vegetables were found across countries. For example, women in our study consumed greater amounts of vegetables than those in China and Thailand [13,47] but had a lower intake of fruit compared with Chinese pregnant women [13]. Particularly, leafy vegetables and fruit-vegetables were the main vegetables consumed in our study. In addition, Vietnamese pregnant women consumed less soy products, poultry, and eggs than Chinese women [13]. The intake levels of animal-based foods were greater in higher-educated women and lower in younger and overweight women when compared to the respective lower groups. These findings are useful for developing guidelines for the population to achieve a balanced diet during pregnancy.

The mean energy intake of 2004 kcal/day in our study met the RNI for pregnant women with light physical activity but failed to attain the RNI for those with moderate physical activity [38]. This intake was similar to estimates from previous studies in low- and middle-income countries [7] and China [14,48], but much higher than the intakes determined in Thailand and India [12,49]. However, this calorie intake was lower than that of pregnant women in some Western countries such as the United States and Canada (2201 kcal/day), Europe (2197 kcal/day), and Australia and New Zealand (2212 kcal/day) [50]. The mean energy intake reported by our participants was higher than that of Vietnamese adults from the general nutrition survey 2009–2010 (1925 kcal/day per capita) [27], but lower than that of WRA (2196 kcal/day) [51]. Notably, half of the study participants did not meet the RNI for energy requirements during pregnancy. In addition, there was a large variation in energy intakes, which may be due to different dietary patterns between North and South Vietnam, seasonal food availability, and individual physical activity levels.

Our study found that protein, fat, and carbohydrate intakes accounted for 15.9%, 31.8%, and 52.2% of total energy, respectively, which are similar to the values determined for Chinese women [48] but are much higher than those obtained for Thai women [12]. No information on macronutrient intakes during pregnancy in Vietnam is available, but two studies conducted in adults and WRA reported that the protein intake was similar to that calculated in our study [27,51]. However, our participants consumed more fat and less carbohydrates than those in these studies. White rice was a major source of carbohydrates in the present study. As such, it may not be necessary for this population to achieve 55–65% of energy from carbohydrates, providing that the food sources are of high quality and supply a range of micronutrients with the carbohydrates. Further improvements in dietary diversity are needed to improve and balance the intakes of macronutrients.

The mean intakes of essential micronutrients for pregnant women, such as folate, vitamin D, calcium, iron, and zinc, were far below the RNI. The deficiency of these micronutrient intakes is prevalent in low- and middle-income countries [7,8,12,13,49] and it is not common in developed countries [52,53]. Our findings present the same problem of micronutrient deficiencies in pregnant women as the previous studies of Vietnamese adults and WRA [26,27,28,29,30]. This may be explained by a low intake of micronutrient-rich foods, such as eggs, fish, soy, and dairy products. In addition, our participants consumed foods that usually do not have high bioavailable iron and zinc. Such insufficient micronutrient intakes may influence foetal metabolism, organ growth, development, and function, and chronic diseases later in life [54]. It is noteworthy that the mean intakes of some micronutrients such as vitamin A, vitamin C, vitamin K, thiamin, riboflavin, and pyridoxine achieved the RNI, but a large number of participants (27.7–54.7%) did not meet the recommendations still.

The association between dietary intakes and socio-economic status has been reported in previous studies in different countries and regions [12,14,29,51,55,56,57,58]. People of higher socio-economic status (education, occupation, or income) tend to have higher intakes of energy, macronutrients [14,51,57], and most micronutrients such as vitamin A, B12, C, D, and E, folate, calcium, iron, and zinc [14,29,55,56,57,58]. Our study also indicates that pregnant women with a higher educational level had significantly higher intakes of proteins, vitamin C, E, thiamin, niacin, pantothenic acid, pyridoxine, iron, zinc, selenium, magnesium, potassium, and phosphorus. It is conceivable that higher-educated women are more health-conscious during pregnancy and thus they may attempt to maintain a high-quality diet. Similarly, pregnant women aged <25 years had significantly higher levels of energy, macronutrients, B vitamins, iron, zinc, and selenium than older women. Moreover, consistent with studies in China and Germany [48,59], pregnant women with a higher pre-pregnancy BMI in our study had significantly lower intakes of energy, macronutrients, and most micronutrients than their counterparts in the lower BMI groups. Probably, overweight women tended to receive advice on the prevention of excessive gestational weight gain through diet control. Alternatively, a lower energy intake in women with pre-pregnancy overweight may be attributed to their lower physical activity levels compared to women without pre-pregnancy overweight and thus the need of less energy to achieve homeostasis. The difference in the average nutrient intakes across key demographic factors and maternal pre-pregnancy weight status is integral to planning interventions targeted at specific groups.

The strengths of the present study include a relatively large sample size from which the typical diet of pregnant women in Vietnam can be extrapolated. Additionally, the dietary intakes were comprehensively analyzed and reported, including food groups, energy, macronutrients, and micronutrients, based on an interviewer-administered food frequency questionnaire and food composition tables for local diet. However, the study has some limitations. The common drawbacks of the FFQ method in epidemiological studies include a recall bias, the selection of core foods, and the lack of information about the preparation methods, that may lead to imprecise estimates of nutrients [60,61]. In the Vietnamese context, there may also be a possible influence of seasonal variation in food sources that makes it difficult to correctly estimate the habitual nutrient intake of pregnant women. Another concern is that Vietnamese women normally share dishes with their family members, which may affect individual portion size estimates. We attempted to minimize these potential errors by asking a comprehensive item food list in Vietnam and conducting direct interviews using supportive materials such as standardized tableware sets and food portion images. Besides, we did not take into account dietary supplement data collected from our participants in the final analysis because it is beyond the scope of this study. Although our preliminary analyses showed that the supplementation rates of zinc, folic acid, multivitamin, iron, and calcium were 2.0%, 8.7%, 28.7%, 85.4%, and 86.0%, respectively (full data not presented for brevity), this is unlikely to have a significant impact on the results.

## 5. Conclusions

In conclusion, the present study indicates low dietary nutrient intakes among pregnant women in Vietnam. The prevalence of mothers failing to meet the national recommendations for essential micronutrient intakes was on average over 50%. Our report provides timely and important data to inform policy makers, researchers, and community stakeholders, so that appropriate nutrition interventions can be implemented to improve the diet quality and the overall health of Vietnamese women during pregnancy.

## Figures and Tables

**Table 1:** Socio-demographic characteristics of the study participants.

Characteristic	*n*	%
Age (years)		
<25	611	31.4
25–29	673	34.6
30–34	450	23.2
≥35	210	10.8
Educational level		
Less than high school	697	35.9
High school	492	25.3
Further than high school	755	38.8
Parity		
0	768	39.5
1	708	36.4
≥2	468	24.1
Pre-pregnancy BMI (kg/m^2^) ^a^		
Underweight, <18.5 kg/m^2^	494	25.4
Normal, 18.5≤BMI<23.0 kg/m^2^	1199	61.7
Overweight, ≥23.0 kg/m^2^	251	12.9
Smoking during pregnancy		
Active smoking	0	0.0
Passive smoking ^b^	1017	52.3
Alcohol drinking during pregnancy ^c^	256	13.2

BMI: Body mass index. ^a^ Based on cut-off for Asian population [40]. ^b^ Defined as any exposure to smoking at home or workplaces during pregnancy. ^c^ Defined as drinking any alcohol during pregnancy.

**Table 2:** Average food intake by pregnant women in Vietnam, 2015–2016.

Food Group (g/day)	Mean ± SD
Cereals	682.8 ± 274.6
Bread	19.0 ± 22.4
Noodle	58.0 ± 48.1
Rice	586.6 ± 274.9
Vegetables	240.7 ± 179.1
Leafy vegetables	87.6 ± 76.4
Roots & tubers	48.2 ± 60.7
Flowers & stems	3.8 ± 8.8
Fruit-vegetables	78.4 ± 82.0
Pulses & sprouts	16.3 ± 27.4
Other vegetables	2.3 ± 7.4
Pickled vegetables	4.2 ± 12.1
Fruits	315.0 ± 235.9
Banana	63.4 ± 89.8
Mango	46.4 ± 63.4
Grapefruit and orange	45.9 ± 74.7
Watermelon	42.5 ± 70.5
Guava	38.6 ± 60.0
Papaya	28.6 ± 62.6
Apple and pear	16.4 ± 31.6
Grape	10.2 ± 26.2
Other fruits	23.0 ± 53.1
Soy products	54.8 ± 94.9
Red meat	46.4 ± 37.9
Poultry	17.6 ± 21.2
Fish & seafood	32.5 ± 36.6
Eggs	26.1 ± 29.0
Dairy	94.4 ± 155.1
Alcohol (grams ethanol/day) *	0.91 ± 1.54
Coffee (cup/day) *	0.37 ± 0.55
Tea (cup/day) *	0.95 ± 1.78
Sweet dessert	35.3 ± 43.2
Fruit juices & soft drinks (mL/day)	127.1 ± 160.7

* The data were obtained from the drinkers, noting that a small number of women drank alcohol (13.0%), coffee (21.9%), and tea (31.3%) during their pregnancy. Alcohol consumption was calculated only for mothers who consumed alcohol during pregnancy.

**Table 3:** Food intake by age group, education level, and pre-pregnancy BMI level among pregnant women in Vietnam, 2015–2016.

Food Group (g/day)	Age (years)					Education Level				Pre-Pregnancy BMI (kg/m^2^)			
	<25	25–29	30–34	≥35	*p*	Less than HS	High School	Post HS	*p*	<18.5	18.5 ≤ BMI < 23.0	≥23.0	*p*
Cereals	707.9	682.5	659.9 ^a^	659.5 ^a^	0.021	678.1	688.8	683.2	0.802	737.1	676.7 ^a^	604.6 ^a,b^	<0.001
Vegetables	226.2	241.1	244.2	274.5 ^a^	0.009	242.9	242.9	237.4	0.804	238.8	244.5	226.7	0.347
Fruits	337.7	301.9 ^a^	306.2	309.9	0.037	299.2	324.8	323.2	0.087	329.8	315.0	285.9 ^a^	0.056
Soy products	51.3	55.4	59.3	53.8	0.595	52.5	59.4	54.0	0.441	46.6	56.4	63.8 ^a^	0.042
Red meat	48.7	46.5	45.9	40.1 ^a^	0.045	43.0	43.1	51.6 ^a,b^	<0.001	49.6	45.6	43.8	0.076
Poultry	20.2	16.6 ^a^	16.6 ^a^	15.4 ^a^	0.003	15.0	17.6	20.0 ^a^	<0.001	19.6	17.4	14.8 ^a^	0.012
Fish & seafood	27.4	34.8 ^a^	34.7 ^a^	35.1 ^a^	0.001	34.4	28.1 ^a^	33.5 ^b^	0.008	34.0	30.9	37.0 ^b^	0.028
Eggs	30.5	25.8 ^a^	21.2 ^a,b^	24.6 ^a^	<0.001	19.9	29.0 ^a^	29.9 ^a^	<0.001	27.7	26.7 ^a^	20.2 ^b^	0.002
Dairy	86.0	102.7	98.8	83.1	0.158	100.5	84.8	95.1	0.224	87.0	95.6	103.2	0.369
Sweet dessert	40.7	34.4 ^a^	32.3 ^a^	29.4 ^a^	0.001	36.6	35.5	34.1	0.528	38.4	34.2	34.7	0.190
Fruit juices & soft drinks (mL/day)	135.4	134.3	120.7	93.8 ^a,b^	0.005	116.7	120.9	140.8 ^a^	0.011	127.9	129.0	116.8	0.549

HS: High school; BMI: Body mass index, using cut-off for Asian population [40]. Data are means; superscript letters indicate a statistically significant difference (*p* < 0.05) according to the Tukey–Kramer test [43]. ^a^ Comparison between that group and “age < 25 years”, “less than HS”, and “pre-pregnancy BMI < 18.5” for age, education level, and pre-pregnancy BMI, respectively. ^b^ Comparison between that group and “age 25–29 years”, “high school” and “pre-pregnancy normal BMI” for age, education level, and pre-pregnancy BMI, respectively.

**Table 4:** Daily energy and nutrient intakes of pregnant women in Vietnam, 2015–2016.

Energy and Nutrient (unit/day)	RNI			Our Study		
	Vietnam MOH	NIH 2016	WHO/FAO 2004	Observed Intakes ^a^	Energy-Adjusted Intakes ^a^	% Meeting Vietnam RNI ^b^
Energy (kcal)	1980–2010	NA	NA	2004 ± 625	-	49.1
Percentage of energy from proteins (%)	13–20	10–35	NA	15.9	-	-
Percentage of energy from fat (%)	20–30	20–35	NA	31.8	-	-
Percentage of energy from carbohydrates (%)	55–65	45–65	NA	52.2	-	-
Protein (g)	70	71	NA	79.4 ± 25.0	40.0 ± 5.2	62.9
Fat (g)	52.5–64.5	NA	NA	70.9 ± 24.2	35.2 ± 4.2	-
Carbohydrate (g)	325–400	175	NA	261.6 ± 85.7	130.5 ± 11.8	-
Fiber (g)	28	28	NA	16.1 ± 6.7	8.1 ± 2.4	-
Vitamin A (µg) ^c^	650–700	770	800	849.7 ± 500.1	431.6 ± 225.1	58.7
Vitamin C (mg)	110	85	55	183.4 ± 118.8	94.1 ± 56.4	72.3
Thiamin (mg)	1.2–1.3	1.4	1.4	1.4 ± 0.5	0.7 ± 0.2	65.0
Riboflavin (mg)	1.5	1.4	1.4	1.5 ± 0.6	0.8 ± 0.2	45.5
Niacin (mg)	18	18	18	26.6 ± 9.4	13.3 ± 2.3	83.2
Pantothenic acid (mg)	6	6	6	5.6 ± 1.9	2.9 ± 0.6	38.0
Pyridoxine (mg)	1.9	1.9	1.9	2.4 ± 0.9	1.2 ± 0.3	71.0
Folate (µg) ^d^	600	600	600	440.8 ± 167.6	224.2 ± 62.1	15.4
Cobalamin (mg)	2.6	2.6	2.6	4.4 ± 1.7	2.2 ± 0.6	88.5
Vitamin D (µg)	15	15	5	2.3 ± 2.2	1.2 ± 1.1	-
Vitamin E (mg)	6.5	15	NA	4.2 ± 1.7	2.1 ± 0.7	9.1
Vitamin K (µg)	150	90	55	267.8 ± 229.5	137.0 ± 108.1	68.2
Calcium (mg)	1200	1000	1200	509.8 ± 263.5	260.2 ± 117.8	2.5
Phosphorus (mg)	700	700	NA	1322.6 ± 447.8	665.4 ± 113.7	94.1
Potassium (mg)	>3510	4700	NA	3038.4 ± 1186.9	1550.8 ± 471.9	29.6
Sodium (mg)	<2000	1500	NA	3312.7 ± 1273.7	1657.8 ± 375.3	12.2
Magnesium (mg)	40	350–360	220	289.1 ± 105.0	147.5 ± 39.3	100
Iron (mg) ^e^	27.4	27	24.5	9.4 ± 3.4	4.8 ± 1.3	0.05
Zinc (mg) ^f^	10	11	11–14	7.7 ± 2.9	3.9 ± 1.1	18.0
Copper (µg)	1000	1000	NA	1.0 ± 0.4	0.5 ± 0.2	0.0
Selenium (µg)	28	60	28–30	118.1 ± 43.0	60.0 ± 15.0	100
Manganese (mg)	2.0	2.0	NA	3.0 ± 1.1	1.5 ± 0.4	84.4

RNI: Recommended nutrient intakes; MOH: Ministry of Health; NIH: National Institutes of Health: WHO: World Health Organization; FAO: Food and Agriculture Organization of the United Nations; SD: Standard deviation; NA: Not available. ^a^ Data are presented as mean ± SD. Energy intakes were adjusted for the amount of nutrient intake per 1000 kcal of energy [45]. ^b^ Based on energy and nutrient intakes compared with the RNI for Vietnamese pregnant women [38]. ^c^ As retinol activity equivalents (RAEs). ^d^ As dietary folate equivalents (DFE). ^e^ Based on the assumption of high bioavailability of iron from the Vietnam diet (15%) [38]. ^f^ Based on the assumption of medium bioavailability of zinc from the Vietnam diet (30%) [38].

## Supplementary Materials

The following are available online at http://www.mdpi.com/2072-6643/10/8/1025/s1, Table S1: Comparison of nutrient intakes by selected characteristics of pregnant women in Vietnam, 2015–2016.
