# Supplementary material for: Low Dietary Intakes of Essential Nutrients during Pregnancy in Vietnam
Source: Nutrients. 2018 Aug 6;10(8):1025. doi: 10.3390/nu10081025 (PMC6116189; doi:10.3390/nu10081025)
Supplement: Supplementary file 1 [file nutrients-10-01025-s001.pdf]

Supplementary Table S1. Comparison of nutrient intakes by selected characteristics of pregnant women in Vietnam, 2015-2016.

Nutrient (unit/day)	Age (years)					Educational level				Pre-pregnancy BMI[a]			
	<25	25-29	30-34	≥35	p	Less than HS	High school	Post HS	p	<18.5	18.5-<23.0	≥23.0	p
Energy (kcal)	2085	2001	1936	1923	<0.001	1977	2006	2026	0.330	2134	1988	1824	<0.001
Protein (g)	81.5	79.8	77.2	76.3	0.011	77.6	78.5	81.5	0.009	83.9	78.6	74.1	<0.001
Fat (g)	73.5	70.9	68.7	67.7	0.002	69.5	70.8	72.2	0.117	75.7	70.3	63.9	<0.001
Carbohydrate (g)	273.7	260.3	251.7	251.7	<0.001	259.8	263.3	262.1	0.763	278.8	259.5	237.6	<0.001
Fibre (g)	16.7	15.9	15.8	16.1	0.126	16.0	16.4	16.1	0.597	16.9	16.2	14.4	<0.001
Vitamin A (μg)[b]	806.5	869.3	862.2	885.7	0.073	856.2	856.6	839.1	0.759	855.9	857.8	798.9	0.226
Vitamin C (mg)	184.6	183.6	180.3	185.6	0.931	175.5	182.7	191.1	0.043	183.0	184.4	179.1	0.809
Thiamin (mg)	1.5	1.4	1.4	1.3	<0.001	1.4	1.4	1.5	0.008	1.5	1.4	1.3	<0.001
Riboflavin (mg)	1.6	1.5	1.5	1.4	0.001	1.5	1.5	1.6	0.058	1.6	1.5	1.5	0.021
Niacin (mg)	28.0	26.5	25.8	24.9	<0.001	25.7	26.7	27.4	0.003	28.5	26.5	23.9	<0.001
Pantothenic acid (mg)	5.8	5.7	5.5	5.4	0.016	5.5	5.7	5.8	0.038	5.9	5.6	5.3	<0.001
Pyridoxyl (mg)	2.5	2.4	2.4	2.4	0.021	2.4	2.4	2.5	0.012	2.6	2.4	2.2	<0.001
Folate (μg)[c]	448.1	443.2	429.5	436.2	0.321	432.6	442.6	447.2	0.243	458.6	438.9	415.3	0.003
Cobalamin (mg)	4.6	4.5	4.4	4.2	0.025	4.3	4.4	4.5	0.113	4.6	4.4	4.2	0.003
Vitamin D (μg)	2.1	2.4	2.3	2.1	0.057	2.3	2.1	2.3	0.129	2.2	2.2	2.4	0.412
Vitamin E (mg)	4.4	4.2	4.0	4.2	0.015	4.1	4.3	4.2	0.073	4.4	4.2	3.9	<0.001
Vitamin K (μg)	265.7	264.4	263.0	295.5	0.325	256.3	275.8	273.3	0.246	276.6	271.3	233.9	0.039
Ca (mg)	509.5	520.3	505.6	486.3	0.416	499.7	508.0	520.3	0.326	511.8	510.2	503.9	0.923
P (mg)	1376	1328	1280	1242	<0.001	1285	1330	1353	0.013	1391	1316	1220	<0.001
K (mg)	3079	3056	2994	2957	0.478	2932	3032	3141	0.004	3137	3042	2829	0.004
Na (mg)	3417	3331	3187	3221	0.021	3227	3316	3389	0.054	3519	3288	3021	<0.001
Mg (mg)	294.2	292.0	283.2	277.9	0.123	279.1	287.9	299.3	0.001	299.5	289.1	268.9	0.001
Fe (mg)[d]	9.7	9.4	9.2	9.0	0.012	9.0	9.5	9.8	<0.001	9.8	9.4	8.9	0.002
Zn (mg)[e]	8.1	7.7	7.5	7.2	<0.001	7.4	7.8	8.0	<0.001	8.0	7.7	7.2	0.003
Cu (μg)	1.0	1.0	1.0	1.0	0.699	1.0	1.0	1.0	0.209	1.0	1.0	1.0	0.123
Se (μg)	122.9	118.5	114.8	109.8	<0.001	112.8	117.7	123.3	<0.001	124.6	117.2	109.9	<0.001
Mn (mg)	3.1	3.1	3.0	3.0	0.425	3.0	3.0	3.1	0.474	3.2	3.0	2.8	<0.001

Data are presented as mean values and based on absolute intakes

HS: High school; BMI: Body mass index
[a] Based on cut-off for Asian population
[b] As retinol activity equivalents (RAEs)
[c] As dietary folate equivalents (DFE)
[d] Based on assumption of high bioavailability of iron from Vietnam diet (15%)
[e] Based on assumption of medium bioavailability of zinc from Vietnam diet (30%)
